# Investigating the role of capacitive coupling between the operating table and the return electrode of an electrosurgery unit in the modification of the current density distribution within the patients’ body

**DOI:** 10.1186/1475-925X-12-80

**Published:** 2013-08-12

**Authors:** Paolo Bifulco, Rita Massa, Mario Cesarelli, Maria Romano, Antonio Fratini, Gaetano D Gargiulo, Alistair L McEwan

**Affiliations:** 1 Department of Electrical Engineering and Information Technology, University Federico II of Naples, Naples, Italy; 2 Department of Physics, University Federico II of Naples, Naples, Italy; 3 School of Electrical and Information Engineering, The University of Sydney, Darlington, New South Wales, Australia; 4 The MARCS Institute, BENS group, The University of Western Sydney, Penrith, New South Wales, Australia

**Keywords:** Electrosurgery, Finite Difference Time Domain (FDTD) electromagnetic analysis, Detailed human tissues 3D model, Operating table, Capacitive coupling

## Abstract

**Background:**

Electrosurgery units are widely employed in modern surgery. Advances in technology have enhanced the safety of these devices, nevertheless, accidental burns are still regularly reported. This study focuses on possible causes of sacral burns as complication of the use of electrosurgery. Burns are caused by local densifications of the current, but the actual pathway of current within patient’s body is unknown. Numerical electromagnetic analysis can help in understanding the issue.

**Methods:**

To this aim, an accurate heterogeneous model of human body (including seventy-seven different tissues), electrosurgery electrodes, operating table and mattress was build to resemble a typical surgery condition. The patient lays supine on the mattress with the active electrode placed onto the thorax and the return electrode on his back. Common operating frequencies of electrosurgery units were considered. Finite Difference Time Domain electromagnetic analysis was carried out to compute the spatial distribution of current density within the patient’s body. A differential analysis by changing the electrical properties of the operating table from a conductor to an insulator was also performed.

**Results:**

Results revealed that distributed capacitive coupling between patient body and the conductive operating table offers an alternative path to the electrosurgery current. The patient’s anatomy, the positioning and the different electromagnetic properties of tissues promote a densification of the current at the head and sacral region. In particular, high values of current density were located behind the sacral bone and beneath the skin. This did not occur in the case of non-conductive operating table.

**Conclusion:**

Results of the simulation highlight the role played from capacitive couplings between the return electrode and the conductive operating table. The concentration of current density may result in an undesired rise in temperature, originating burns in body region far from the electrodes. This outcome is concordant with the type of surgery-related sacral burns reported in literature. Such burns cannot be immediately detected after surgery, but appear later and can be confused with bedsores. In addition, the dosimetric analysis suggests that reducing the capacity coupling between the return electrode and the operating table can decrease or avoid this problem.

## Background

Electrosurgery unit (ESU) operation assumes that current densities within patient’s body are high only at the point of contact with the active electrode and are much lower in other tissues, in order to produce heating effect only at that contact point [1].

The modern ESU is a floating applied part (usually type BF [2]) with no direct path to ground. This reduces hazards inherent in grounded systems such as current division and alternate site burns [3-6]. Electronic continuous monitoring of the proper connection of the dispersive electrode and its contact with patient’s skin add further safety features.

While the monitoring of currents within the electrodes and cables is easily to achieve, it is very difficult to predict the exact current path within the patient body.

The human body is a high loss non-homogeneous medium for electrical currents and local dielectric properties depend on the type of tissue. Hence, current does not flow uniformly through patient’s body but tends to preferably flow through low impedance tissues. Moreover, capacitive couplings and/or alternate return paths may also occur, due to surgical instrumentations (e.g. surgery instruments, laparoscopy tools), other conductive parts (e.g. a metal operating table, electrodes) or patient’s prosthesis. As a consequence, risks of unintentional tissue damage, including burns (known as alternate site burns), exist and possible causes must be investigated and prevented.

Since the temperature rise in tissues is primary related to the current density [7,8], a detailed analysis of the spatial distribution of current density inside the patient’s body is needed.

This necessarily requires the knowledge of individual patient’s anatomy, electrical properties of various tissues, body positioning with respect to the applied electrodes of ESU and the operating table [9,10].

In particular, this study was motivated by some evidences of suspicious sores in sacral region of patients who had undergone chest surgery supported by monopolar ESU. Generally, the patient laid supine on the operating table and the return electrode was placed on his back near the shoulder blade. The ESUs used in surgery were correctly functioning and passed all the electrical safety and functioning tests required by the international standards [2,11]. Because of the location of the sores (i.e. quite far from the applied electrodes), these lesions were not promptly associated to the use of ESU. Further, the incidence of sores was dramatically decreased by placing an additional insulating pad between the return electrode and the mattress of the operating table. This evidence suggests possible capacitive couplings between the return electrode and the metallic structure of the operating table.

Similar problems are reported by various studies available in the literature [12-15], which mention that the sacral region may be subjected to sores and inflammation after surgery that uses electrosurgical units. These kind of burns, which originate beneath patient’s skin, are not usually detected immediately after surgery in the operating theater but instead some days later. Usually, surgeons do not notice irregularities during surgery indicating the possibility of electrode burns and therefore these burns are often wrongly diagnosed as bedsores.

The aim of this study is to analyze, in detail, current densities inside patient’s body so to provide information about their thermal effect, during the ESU operation, by means of a detailed and realistic representation of the surgical environment (i.e. the patient’s body, operating table, mattress, and electrodes) and a Finite Difference Time Domain (FDTD) electromagnetic analysis. To this end, we adopted the detailed anatomical model of a whole human body provided by SPEAG (Virtual Family) that accurately represents seventy-seven different tissues and their electrical properties, and can be easily imported in the SEMCAD solver, in the belief that the results of the FDTD analysis can shed a light on practical situations that can arise in clinical setting.

## Methods

A detailed, anatomical, 3D whole-body model of an adult, based on high-resolution MR images, in which seventy-seven tissues and organs are represented and associated with their corresponding electrical properties, was considered [16-19]. The spatial resolution of 2 mm was chosen for the model. Appropriate values of electrical properties (i.e. relative permittivity, magnetic permeability and conductivity) were assigned to each tissue when the frequency was set [16].

The operating table and electrodes of the ESU (both active and return) were considered as perfect electric conductors, because their actual conductivity is some orders of magnitude higher than the other materials considered. The operating table was considered to be at the ground potential to resemble a real situation. The electrical properties of the mattress at the ESU working frequencies were chosen in accordance with a previous study that considered a typical mattress in polyurethane for operating table: relative permittivity ϵ_r_ = 50 and conductivity σ = 10^-4^ (S/m) [20].

In order to resemble a real situation, the patient was set supine on the mattress of the operating table; the return electrode (about 300 cm^2^) was placed, perfectly attached, to his back and the active electrode was placed on the anterior part of the thorax. A sinusoidal peak-to-peak voltage of 1000 V was applied between the electrodes [7]; as the ESU applied part is floating this voltage was distributed symmetrically with respect to the earth ground. Different operating frequencies were considered (i.e. within the range 400 kHz and 5 MHz), which are typical of most electrosurgical devices.

At these frequencies, the wavelength of electromagnetic field is much larger than the system size. Therefore, as for most electrosurgery simulations, it was sufficient to reduce the Maxwell equations to a form similar to electrostatics (quasi-static approximation) [9]. Most of the energy generated by the ESU device is dissipated through electrical conduction rather than capacitive coupling.

The quasi-static approximation is justified if the following relationship is verified

(1) $$L<<\lambda \;\Rightarrow \;\mathrm{Lf}<<\frac{1}{\sqrt{\epsilon \mu }}$$

where *L* is the system size, *λ* the wavelength, *f* the frequency of the electromagnetic wave, ϵ and *μ* the permeability and permittivity of the medium. This relationship is largely verified by considering the operating frequencies of the ESU devices, the electromagnetic properties of the human tissue and their size.

When the quasi-static approximation holds, the fundamental equations for the numerical simulation for these low-frequency fields in conductive materials are the same as that for electrostatics. Therefore, all our simulations were obtained by setting the quasi-static approximation option for the SEMCAD (Schmid and Partner Engineering AG, Switzerland) solver.

## Results

**Figure 1:**
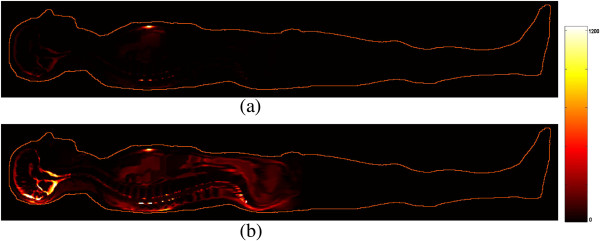
**Current density distribution (A/m**^**2**^**) within patient’s body (mid-sagittal section) resulted by the FDTD analysis: (a) with a non-conductive operating table; (b) with a conductive operating table considered at ground potential.** The active electrode is located about at sternum (note the high current-density spots in both figures) while the return electrode is at the patient’s back. For the reported example a 400 kHz frequency is assumed.

The mid-sagittal sections of the patient are shown in Figure 1 and represent the distribution of the modulus (amplitude) of the current density inside patient’s body. Numerical simulations were performed considering the operating table non-conductive (a) and conductive (b).

As expected, in the case of non-conductive operating table the current is confined well between the active and passive electrodes. On the contrary, if the operating table is considered to be conductive the current is distributed in a much more extended way within patient’s body, taking advantage of the effects of coupling with the metal table. High concentrations of current are visible in the low impedance areas of the patient’s head and behind the sacrum.

**Figure 2:**
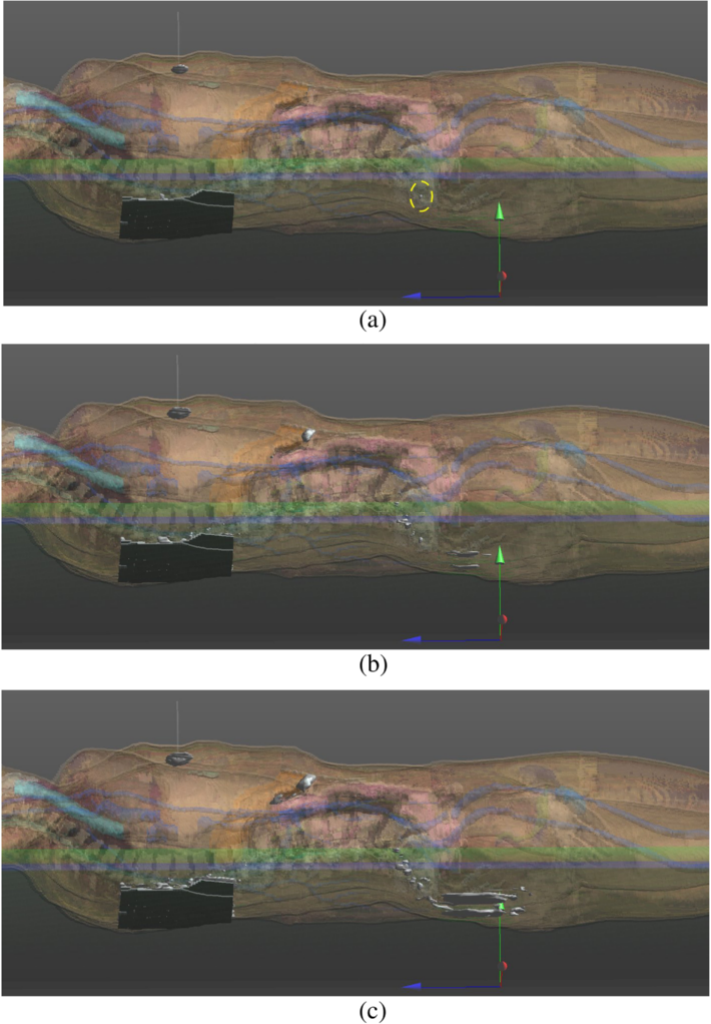
**3D views of isosurfaces obtained at different current density thresholds in the case of conductive table: (a) 1500 A/m**^**2**^**; (b) 800 A/m**^**2**^**; (c) 600 A/m**^**2**^**.** The isosurfaces are colored in gray and represented as opaque, while the different tissues of the patient assume different colors and are represented as semi-transparent. The right lateral edges of the mattress and the operating table appear as green and blue horizontal lines. In **(a)** a yellow dashed circle surrounds the small isosurface located just behind the sacrum.

Figure 2 presents the results obtained by the numerical simulations in the case of conductive table by means of a three-dimensional rendering. This helps in understanding the spatial distribution of the current within patient’s body. 3D isosurfaces, were computed where the current density was higher than preset thresholds (i.e. 1500, 800 and 600 A/m^2^). The isosurfaces are represented in gray and are superimposed to the patient’s body where different colors represent different tissues. The active electrode appears as a thin line normal to the thorax (in correspondence of the highest current density region) and the return electrodes appear highlighted on the patient’s back. It is important to note that also above the threshold of 1500 A/m^2^, a small spherical spot is present just behind the sacrum (a yellow dashed circle highlights it). At a lower threshold (600 A/m^2^), the current density area is clearly enlarged behind the sacrum and on the upper part of the buttocks. This is very compatible with actual postoperative burns reported by other authors [12-15].

The difference of current density in both cases (with and without the conductive table) was considered indicative of the influence of a conductive table to the ESU current distribution within the patient’s body.

**Figure 3:**
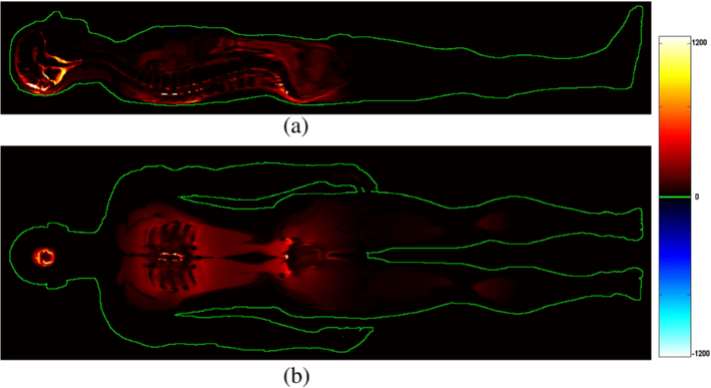
**Sagittal (a) and coronal (b) sections of the patient representing the difference between the two current densities (A/m**^**2**^**) (with and without conductive table).** A high current density spot is evident behind the sacrum. High values of current densities results also in the patient head and in correspondence of the thoracic intervertebral discs (much more conductive than vertebrae).

Figure 3 shows the results obtained by computing the difference of the current densities for each sampling element (a mid-sagittal section (a) and a coronal section at about the level of the shoulder (b) are shown).

### Sensitivity analysis

A sensitivity analysis was performed to verify the dependence of the results on some of the electromagnetic properties of the materials involved. Since the conductive table and the two electrodes of the ESU are metallic, the mattress is the only additional material whose properties may vary. Hence, the parameters characterizing the dielectric material of the mattress were varied to test their influence on the results of the FDTD simulation. In particular the relative permittivity was varied within the range 1–100 and the electrical conductivity between 0-10^-4^ (S/m) (all previous simulations assumed relative permittivity equal to 50 and conductivity equal to 10^-4^ (S/m)).

Since we are particularly interested in the sacral region, the following Table 1 summarizes the maximum difference ∆J_max_ of current density computed in a region centered at the sacral bone that extended to a radius of 10 cm (between the conductive-table and non-conductive-table cases) resulting from changes of the electromagnetic properties of the mattress.

**Table 1:** Max differences of current density between the conductive-table and non-conductive-table cases within the lumbosacral region, obtained by varying the dielectric parameters of the mattress (conductivity and relative permittivity)

**∆J**_**max**_	**ϵ**_**r**_ **= 1**	**ϵ**_**r**_ **= 5**	**ϵ**_**r**_ **= 20**	**ϵ**_**r**_ **= 50**	**ϵ**_**r**_ **= 100**
σ = 10^-4^ (S/m)	+247 (A/m^2^)	+645 (A/m^2^)	+1491 (A/m^2^)	+2492 (A/m^2^)	+3418 (A/m^2^)
σ = 0 (S/m)	+247 (A/m^2^)	+645 (A/m^2^)	+1491 (A/m^2^)	+2492 (A/m^2^)	+3418 (A/m^2^)

All data are expressed as A/m^2^, the positive sign means that current density is increased with respect to the non-conductive-table case.

As expected, the current densities increase as the relative permittivity of the mattress increases. No appreciable variation (i.e. less than 1 A/m^2^) resulted by varying the conductivity of the mattress. These results support the simplifying hypothesis that capacitive coupling sustain the increase of the patient’s currents outside the primary path (straight connections between the active and the return electrode).

Since almost all electrosurgical units available on market operate with frequencies between the hundreds of kHz and a few MHz, the numerical simulation was repeated by considering the following frequencies: 400 kHz (that used for all other simulations), 800 kHz, 1.2 MHz, 5 MHz, to test possible differences depending on frequency. Results revealed a substantial independence of the values of current density inside the patient to frequency. The average difference of current densities over the whole test volume (whole body of the patient) was found to be less than 1 A/m^2^ for all the frequencies considered, while the maximum difference was 31 A/m^2^.

### Heat of tissues

High level of current density lead to increases in temperature within patient’s body. More precisely, the rise in temperature for tissues traversed by electric current can be represented by the simplified Bioheat equation [7,8].

(2) $$\mathrm{\Delta T}=\frac{1}{\mathrm{\sigma \rho c}}J^{2}t$$

If heat transfer to surrounding tissues, blood perfusion and metabolic heat can be neglected, then rise in temperature results directly proportional to the square of the current density (J) and the duration of the application of the current (t) and inversely proportional to the specific heat of the tissue (c), the electrical conductivity (σ) and the tissue density (ρ).

Hence, the increase in temperature of the tissues depends on the time of use of the ESU. This mainly depends on the type of surgery, where open chest cardiac surgery usually requires a long duration of up to several hours, the specific complexity of the intervention and any periods of pause in application of current. These variables depend on the specific surgery and therefore estimation of tissue heating needs accurate case-by-case knowledge. Overall, any prolonged use electrosurgery increases the risk of burns.

In order to provide quantitative information, the time of continuous usage of the ESU needed to increase of 10°C the temperature of a small volume of about 1.5 cm^3^ behind the sacral bone was computed accordingly to the Bioheat equation (i.e. equation 2). The volume was opportunely centered at the local maximum of current density obtained by the FDTD analysis (please, refer to the Figure 2a: the point surrounded by the dashed yellow circle). The time to obtain a temperature rising of 10°C (e.g. from 37°C to 47°C) was computed by considering different ESU power levels [7] and also different relative permittivities of the mattress. Table 2 summarizes these findings for the case of a conductive operating table.

**Table 2:** Estimated time (in seconds) of continuous operation of ESU in cut mode needed to increase the temperature of 10°C at the sacrum (average on a volume of about 1.5 cm3)

**Time to rise temperature of 10°C at sacrum**	**50 W**	**100 W**	**150 W**	**200 W**	**300 W**
ϵ_r_ = 1	5966 (s)	2983 (s)	1989 (s)	1491 (s)	994 (s)
ϵ_r_ = 5	875 (s)	437 (s)	292 (s)	219 (s)	145 (s)
ϵ_r_ = 20	164 (s)	82 (s)	55 (s)	41 (s)	27 (s)
ϵ_r_ = 50	59 (s)	29 (s)	19 (s)	15 (s)	*10 (s)*
ϵ_r_ = 100	31 (s)	16 (s)	10 (s)	8 (s)	5 (s)

Time was estimated by means of the Bioheat equation: the assumption of absence of heat transfer to surrounding tissues, blood perfusion and metabolic heat was held. Time was estimated for a continuous sinusoidal waveform (typical of cut-mode); if coagulation or blend waveforms are delivered by the ESU this time should multiplied by opportune factors (e.g. if a blend-mode is obtained with a duty cycle of 50% the values in the table should by multiplied by 2) The value shown in italics matches the ESU’s settings considered for the simulation.

The settings of the ESU considered for the simulation match to an output power of about 300 W and a relative permittivity ϵ_r_ = 50 for the mattress (the corresponding time value is underlined in the table); according to the results of the heating analysis a time of 10 seconds of continuous ESU operation leads to an increase of 10°C on the specified volume. Maintaining the same set-up, but considering the operating table as non-conductive, the time to increase temperature of 10°C changes from 10 seconds to 1568 seconds (about 26 minutes). This confirms again the role of capacitive coupling between a conductive operating table, the return electrode and the patient’s body and the risk of burns.

Results presented in Table 2 refer to continuous operation of ESU, while delivering a sinusoidal waveform. Pure sinusoidal waveforms are usually adopted by ESU for the cut-mode, but to obtain coagulation or blend (a mix of cut and coagulation) modalities, the sinusoid is usually modulated in amplitude. For example, a blend-mode can be obtained by repeatedly switching on and off the ESU output with a duty-cycle of 50%: this modality of functioning (on equal terms with other parameters) will double the times reported in Table 2. Therefore, when using the ESU in blend-mode or in coag-mode a general lengthening of the times of Table 2 should be considered. An exact calculation requires the knowledge of the precise waveforms delivered by the ESU devices, which may change depending on the manufacturer and the model.

Moreover, it should be taken into account that the Bioheat equation reported in equation 2 does not consider the local thermal exchanges and metabolism: heat transfer to surrounding tissues, blood perfusion and metabolic are neglected. This consideration, namely the fact of neglecting the effect of local cooling, leads, in practice, to a further lengthening of the times reported in Table 2 in practical cases.

Another important aspect to consider is the blood perfusion of tissues. The head of the patient is considerably more perfused with blood compared to the sacral region, and tends to cool much faster. In addition, the sacral region of the patient in the supine position places pressure on the mattress, increasing capacitive coupling and reducing the cooling effects.

From the analysis of the results it can be qualitatively concluded that the area of the sacrum can be subjected to a unwanted rise in temperature that may result in postoperative burns.

## Discussion

Modern technologies offer increasing safety for ESU devices. However, it is possible to electronically monitor only current passing into the two electrodes wires, therefore the current density distribution within a patient’s body remains unknown. ESU safety information contains only general warnings about the possibility of unwanted concentration of the current density in tissues [21-23].

**Figure 4:**
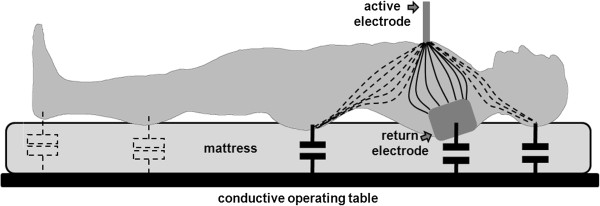
**Qualitative illustration of current distribution inside patient’s body and capacitive coupling with a conductive operating table.** Continuous lines draw the primary current path connecting the two electrodes. The capacity between patient’s body and the table (even if it is distributed) is depicted as concentrated capacitors. Capacitive effects are more relevant where the body closer face the table surface (i.e. head, shoulder and pelvis). The dashed lines represent alternative paths for the current generated by the electro surgical device. Note that all capacitive currents must close through the capacitance coupling of the neutral electrode (which can be opportunely reduced). Local values of the current density inside the patient’s body depends on the specific conductivity of the tissues (e.g. sacrum bone is less conductive than surrounding tissues).

Detailed 3D human model and electromagnetic numerical analysis can provide information about current density distribution within patient’s body. Repeated incidents of sacral burns related to surgical procedures fostered this analysis. A possible occurrence of a concentration of current density in the sacral region, which can generate thermal damage to tissues, was found. When a patient lays supine on operative table, pressure on the mattress results in concentrated current density at specific regions (i.e. head, shoulders and sacral); this makes capacitive coupling with metallic tables more intense than elsewhere. An alternative path for ESU currents is possible outside patient’s body involving the capacitance between the body and table and the capacitance between the table and the return ESU electrode. The capacitive coupling between operating table and patient’s body is dispersed on body surface and so are the currents following this path. However, a simplified description can be provided, by considering distributed capacitance as concentrated only at the return electrode and at the lumbosacral region as depicted in Figure 4. Current inside the patient’s body is also concentrated into more conductive tissues; in particular, at the pelvic region, the relatively lower conductivity of hip bone, sacrum and coccyx forces the current to concentrate in the soft tissues, which become very thin in this region. This circumstance, combined to the fact that capacitive coupling is more intense at the gluteal area (where patient’s body is closer to the table) can qualitatively explain the relatively high value of current density obtained in this analysis.

It is worth underlining that blood circulation in the sacral region is extremely poor compared with other areas of the body, especially when the patient is lying in the supine position and a considerable contact pressure is generated. This impaired circulation results in a reduction in the dispersal of the heat generated in tissues by the current flow induced by the ESU [12].

Surgery-related sacral burns may not appear as superficial burns but deep dermal or full-thickness burns. Since burns are not usually detected immediately after surgery but appear later, they are not always recognized as related to the ESU and can be wrongly diagnosed as bedsores [24,25] or toxic or allergic reactions to the disinfectant solution. In addition, it is difficult to establish whether the burns have been favored by excessive moistening of the operating site, of blood and rinsing fluid that seeped underneath the supine patient (especially about the sacrum) during the operation owing to a leaking or saturated cover, or of urine from a leaking in-dwelling catheter [12,13].

## Conclusions

Electromagnetic simulations performed by using detailed models of patients subjected to surgery are a valuable tool to analyze the distribution of the currents generated by electrosurgical devices within the patient’s body and to disclose their potential risks.

This study provides a plausible explanation to particular burns in the sacral region (extended to the upper part of the buttocks) that sometimes are found in patients who have undergone surgery in which monopolar electrosurgical units were used with high power and for a long time.

The simulation revealed an unexpected high current density located behind the sacrum due to capacity coupling with the conductive operating table. This result has a general implication: although each patient has its own characteristics, the anatomy of the pelvic area promotes the densification of the current in the adjacent subcutaneous soft tissues (posterior to sacral bone) when patient lays supine on a conductive operating table.

The thermal analysis confirms the potential risk of burn due to excessive local heating of those tissues. However, temperature rising in tissues strongly depends on the mode and power setting of ESU and its operating time. These values may vary greatly depending on the particular surgery and should be carefully taken into account.

Multiple strategies may be considered to reduce the abnormal current density in lumbar regions due to capacitive coupling found in this study. The most obvious is that of reducing the capacity between the return electrode and the table: this can be simply achieved by means of interposition of insulating pads or cushions.

The high current density found inside patient’s head, even if it does may not lead to an excessive temperature rises due to blood perfusion, is worthy of more detailed analysis as there are widespread concerns of increased current density or heat generation in neural tissue.

Further analysis should focus on the influence that the individual anatomy of the patient and his posture on the operating table may have with respect to the considered case.

## Abbreviations

3D: Three dimensional; BF: Body floating; ESU: Electrosurgery unit; FDTD: Finite difference time domain; SPEAG: Schmid and partner engineering AG, Switzerland.

## Competing interests

The authors declare that they do not have competing interests.

## Authors’ contributions

All the authors actively participated in the electromagnetic analysis, the processing and interpretation of results and in the preparation of the manuscript. All authors read and approved the final manuscript.

## References

[B1] Jones CM Pierre KB Nicoud IB Stain SC Melvin WV Electrosurgery Curr Surg 2006 63 6 458 463 10.1016/j.cursur.2006.06.017 17084778

[B2] International Electrochemical Commission International standard IEC 60601–1 Medical electrical equipment - Part 1: General requirements for basic safety and essential performance 2012 3 Geneva (with amendment 1)

[B3] Lipscomb GH Givens VM Preventing electrosurgical energy–related injuries Obstet Gynecol Clin North Am 2010 37 3 369 377 10.1016/j.ogc.2010.05.007 20674780

[B4] Neufeld GR Foster KR Electrical impedance properties of the body and the problem of alternate-site burns during electrosurgery Med Instrum 1985 19 2 83 87 4000013

[B5] Emergency Care Research Institute Higher currents, greater risks: preventing patient burns at the return-electrode site during high-current electrosurgical procedures Health Devices 2005 34 8 273 279 16255231

[B6] Vilos G Latendresse K Gan BS Electrophysical properties of electrosurgery and capacitive induced current Am J Surg 2001 182 3 222 225 10.1016/S0002-9610(01)00712-7 11587681

[B7] Eggleston JL Von Maltzahn WW Joseph D Electrosurgical devices The biomedical engineering handbook 2006 3 Bronzino: CRC Press

[B8] Pearce JA Electrosurgery 1986 New York: John Wiley

[B9] Panescu D Whayne JG Fleischman SD Mirotznik MS Swanson DK Webster JG Three-dimensional finite element analysis of current density and temperature distributions during radio-frequency ablation IEEE Trans Biomed Eng 1995 42 9 879 890 10.1109/10.412649 7558062

[B10] Tungjitkusolmun S Je WE Cao H Tsai JZ Vorperian VR Webster JG Finite element analyses of uniform current density electrodes for radio-frequency cardiac ablation IEEE Trans Biomed Eng 2000 47 1 32 40 10.1109/10.817617 10646277

[B11] International Electrochemical Commission International standard IEC 60601-2-2 Medical electrical equipment - Part 2–2: Particular requirements for the basic safety and essential performance of high frequency surgical equipment and high frequency surgical accessories 2009 5 Geneva

[B12] Aigner N Fialka C Fritz A Wruhs O Zoch G Complication in the use of diathermy Burns 1997 23 3 256 264 10.1016/S0305-4179(96)00113-1 9232288

[B13] Vedovato JW Pólvora VP Leonardi DF Burns as a complication of the use of diathermy J Burn Care Rehabil 2004 25 1 120 123 10.1097/01.BCR.0000105047.29499.20 14726750

[B14] Demir E O’Dey DM Pallua N Accidental burns during surgery J Burn Care Res 2006 27 6 895 900 10.1097/01.BCR.0000245650.67130.5C 17091089

[B15] Demirtaş Y Ayhan S Yavuzer R Latifoğlu O Çelebi C Electrosurgery: pitfalls and recommendations Gazi Med J 2006 17 3 27 33

[B16] Christ A The Virtual Family—development of surface-based anatomical models of two adults and two children for dosimetric simulations Phys Med Biol 2010 55 N23 N38 10.1088/0031-9155/55/2/N01 20019402

[B17] Gabriel C Gabriel S Corthout E The dielectric properties of biological tissues: I Literature Phys Med Biol 1996 41 11 2231 2250 10.1088/0031-9155/41/11/001 8938024

[B18] Gabriel C Gabriel S Corthout E The dielectric properties of biological tissues: II. Measurements in the frequency range 10 Hz to 20 GHz Phys Med Biol 1996 41 11 2251 2270 10.1088/0031-9155/41/11/002 8938025

[B19] Gabriel C Gabriel S Corthout E The dielectric properties of biological tissues: III Parametric models for the dielectric spectrum of tissues Phys Med Biol 1996 41 11 2271 2294 10.1088/0031-9155/41/11/003 8938026

[B20] Bellomo L Calí C Vitrotti R Electrical characterization of operating table mattresses Internet J Med Technol 2004 2 1 1 9

[B21] Saye WB Miller W Hertzmann P Electrosurgery thermal injury. Myth or misconception? Surg Laparosc Endosc 1991 1 4 223 228 1669408

[B22] Nduka CC Super PA Monson JR Darzi AW Cause and prevention of electrosurgical injuries in laparoscopy J Am Coll Surg 1994 179 161 170 8044385

[B23] Loffer F Pent D Indication, contraindication and complications of laparoscopy Obstet Gynecol 1975 30 407 427 10.1097/00006254-197507000-00001 124409

[B24] Stewart TP Magnano SJ Burns or pressure ulcers in the surgical patient? Decubitus 1988 1 1 36 40 3254701

[B25] Defloor T De Schuijmer JDS Preventing pressure ulcers: an evaluation of four operating-table mattresses Appl Nurs Res 2000 13 3 134 141 10.1053/apnr.2000.7653 10960997

